# Identification of survival-related alternative splicing signatures in acute myeloid leukemia

**DOI:** 10.1042/BSR20204037

**Published:** 2021-07-20

**Authors:** Biyu Zhang, Lei Yang, Xin Wang, Denggang Fu

**Affiliations:** 1 School of Pharmacy and Life science, Jiujiang University, Jiujiang, Jiangxi 332005, China; 2 School of Biomedical Engineering, Shenzhen University, Shenzhen 518060, China; 3 School of Medicine, Medical University Of South Carolina, Charleston, SC 29425, U.S.A.

**Keywords:** Acute myeloid leukemia, Alternative splicing, Functional enrichment analysis, Prognostic signature, Splicing factor

## Abstract

Aberrant RNA alternative splicing (AS) variants play critical roles in tumorigenesis and prognosis in human cancers. Here, we conducted a comprehensive profiling of aberrant AS events in acute myeloid leukemia (AML). RNA AS profile, including seven AS types, and the percent spliced in (PSI) value for each patient were generated by SpliceSeq using RNA-seq data from TCGA. Univariate followed by multivariate Cox regression analysis were used to identify survival-related AS events and develop the AS signatures. A nomogram was developed, and its predictive efficacy was assessed. About 27,892 AS events and 3,178 events were associated with overall survival (OS) after strict filtering. Parent genes of survival-associated AS events were mainly enriched in leukemia-associated processes including chromatin modification, autophagy, and T-cell receptor signaling pathway. The 10 AS signature based on seven types of AS events showed better efficacy in predicting OS of patients than those built on a single AS event type. The area under curve (AUC) value of the 10 AS signature for 3-year OS was 0.91. Gene set enrichment analysis (GSEA) confirmed that these survival-related AS events contribute to AML progression. Moreover, the nomogram showed good predictive performance for patient's prognosis. Finally, the correlation network of AS variants with splicing factor genes found potential important regulatory genes in AML. The present study presented a systematic analysis of survival-related AS events and developed AS signatures for predicting the patient’s survival. Further studies are needed to validate the signatures in independent AML cohorts and might provide a promising perspective for developing therapeutic targets.

## Introduction

Acute myeloid leukemia (AML) is one of the most aggressive and heterogeneous hematologic malignancies characterized by uncontrolled clonal expansion of poorly differentiated myeloid cells [1,2]. It is estimated that 19,940 new cases and 11,180 new deaths occurred in the US in 2020 [3]. Increasing evidence demonstrated that the course of AML is marked by poor prognosis and recurrent relapse that are closely associated with older age, cytogenetic abnormalities, and genetic mutations. Various investigations have focused on developing novel therapeutics in recent years [4,5], while the overall survival (OS) of AML patients has not significantly improved in the several past decades. Thus, there is an urgent need to identify additional prognostic biomarkers and develop effective therapies to cure AML [1].

RNA alternative splicing (AS) is a critical regulatory process of gene expression post-transcription [6] that contributes to proteome diversity, and functional and phenotypic complexity by generating distinct RNA isoforms from a single gene through different arrangements, including removal of intronic regions and selective inclusion or exclusion of specific exons [7]. Alternative AS events have become a hallmark of cancer, and potential targets for developing new therapeutics [8]. Many AS events have been identified that are correlated with several cancer-related hallmarks, such as epithelial–mesenchymal transition (EMT) [9], apoptosis resistance, invasion and migration, and susceptibility to drug resistance [10]. Transcriptional plasticity controlled by AS events can be employed by cancer cells to produce isoforms that promote cell proliferation or migration. Cis-regulating sequences and splicing factors (SFs) are regarded as important mediators in the process of pre-mRNA maturation. These common cis-regulatory elements can be divided into exonic splicing enhancers, exonic splicing silencers, intronic splicing enhancers, and intronic splicing silencers, each of which has different affinities to SFs. However, trans-acting factors, such as heterogeneous nuclear ribonucleoprotein (hnRNP) protein families, lead to the activation or inhibition of specific splice sites [11]. For example, RBM47, an important RNA-binding protein, was proved to promote EMT by regulating AS of tight junction protein 1 (TJP1) [12] and suppress tumor growth via inhibiting nuclear factor erythroid 2-related factor 2 (NRF2) activity [13] in lung adenocarcinoma. Previous studies proved that the dysregulation of SFs leads to aberrant AS events [14]. The potential regulatory network between SFs and AS events is also imperative to be investigated for discerning important SFs.

In the present study, the comprehensive profiling of genome-wide alternative splicing events of AML cohort from The Cancer Genome Atlas (TCGA) was screened using a strict criterion with SpliceSeq. Survival-related AS events were identified, and the key prognostic AS events selected by the lasso penalized Cox model were used to construct a robust signature for predicting patient's outcome. Functional enrichment analysis of parent genes of survival-related AS events integrated with prognostic SF-AS events network suggests the pathways that have been reported to contribute to AML progression. A prognostic nomogram including clinical parameters was developed to aid in predicting patient survival.

## Materials and methods

### Data acquisition and processing

RNA sequencing data of AML patients along with clinical information were downloaded from TCGA. RNA alternative splicing profiling, including seven AS types, and percent spliced in (PSI) value for each patient was generated by SpliceSeq software. PSI represented the transcript ratio of the parent gene to the type of seven AS events [15]. Strict filter processes were implemented to reliably determine the AS events. The inclusion criteria were as follows: (1) the percentage of samples with PSI value was greater than 80; (2) patients with complete and definitive clinical characteristics including age, gender, FAB subtype, cytogenetics risk category, bone marrow blast cell percent, cellularity percent, and lymphocyte percent; (3) patients who have follow up survival time were enrolled after the initial pathological diagnosis of AML; and (4) patients that have the corresponding RNA-seq splicing variant. Patients with ambiguous features were excluded. Finally, 152 patients were used for further analysis.

Additionally, we used the specific pattern of ‘ABCB9_24994_AP’ to assign each AS event a unique annotation term, in which ‘ABCB9’ represented the parent gene symbol, ‘24994’ stood for the ordered index of this specific AS event in the TCGASpliceSeq database, and ‘AP’ indicated the splicing type.

### Identification of survival-related AS events

To determine the potential clinical prognostic significance of each type AS event, overall survival (OS)-related AS events with *P*<0.05 were identified using univariate Cox proportional hazard regression analysis. Interactive sets between seven types of OS-related AS events were displayed using the UpsetR package [16] in R platform (version 3.6.3).

### Functional enrichment analysis

Parent genes of OS-related AS events were used for functional enrichment analysis by the clusterProfiler package [17]. Gene ontology terms categories including biological process (BP), cellular component (CC), and molecular function (MF), and pathways referenced from the Kyoto Encyclopedia of Genes and Genomes (KEGG) were analyzed. The false discovery rate (FDR) less than 0.05 was considered as statistically significant.

Gene set variation analysis (GSVA) was applied to identify the significantly enriched GO terms and pathways, which were curated annotated gene sets in Molecular Signatures Databases (MSigDB) [18] and parent genes of survival-related AS events using the GSVA package [19]. Differential gene sets in the high-risk group compared to those in the low-risk group were assessed using the limma package [20] with |logFC| > 0.58 and FDR < 0.05.

### Development of the prognostic signature based on AS events for AML patients

The key AS features in each AS type were identified by lasso penalized Cox regression model with 10-fold cross-validation using the top significant AS events. The minimum number of AS events that comprises the final signature was determined by the Akaike information criterion (AIC) [21] to develop the prognostic signature by multivariate Cox stepwise regression analysis. The signature predictive performance was determined by the receiver operating characteristics (ROC) curve using the *survivalROC* package [22]. The risk score for each patient was calculated, and the patients were divided into low- and high-risk groups according to the median risk score. Finally, the prognostic utility of the signature was assessed by the log-rank test.

### Development of an AS-Clinicopathologic Nomogram

To individually predict the survival rate of AML patients, a nomogram incorporating the 10-AS-event-based signature with clinicopathologic variables described above was performed using the rms package [23]. A backward stepwise variable selection with the AIC was used to determine the final nomogram. Then, the decision curve analysis (DCA) was conducted to estimate the clinical utility of the nomogram by quantifying net benefits against a range of threshold probabilities [24].

### Gene set enrichment analysis (GSEA) for the AS signature

To uncover the potential pathways of AS events that are involved in the process of carcinogenesis and progression, GSEA, a computational algorithm that determines the potential statistically significant and concordant differences for a priori defined set of genes in two biological conditions, was implemented with the JAVA program from MSigDB database [18]. The genes were ranked according to differential significance in the annotated ‘C2: curated gene sets’ and ‘C5: GO gene sets’ between low- and high-risk groups. The significant enriched gene set with *P*<0.05 was assessed via 1000 permutations.

### Construction of the correlation network between SFs and AS

The expression profile of SFs genes in the mRNA splicing pathway was extracted from the RNA-seq dataset of AML patients. Spearman correlation matrix between the expression level of OS-associated SFs and PSI values of AS events that were included in the construction of each prognostic signature was analyzed. *P* values were adjusted by Benjamini and Hochberg (BH) correlation. Then, the potential SFs-AS regulatory network was generated among the significant correlation pairs (adjusted *P*<0.05) by Cytoscape (version 3.6.1).

## Results

### Characteristics of AS event profiles in AML cohort

**Figure 1:**
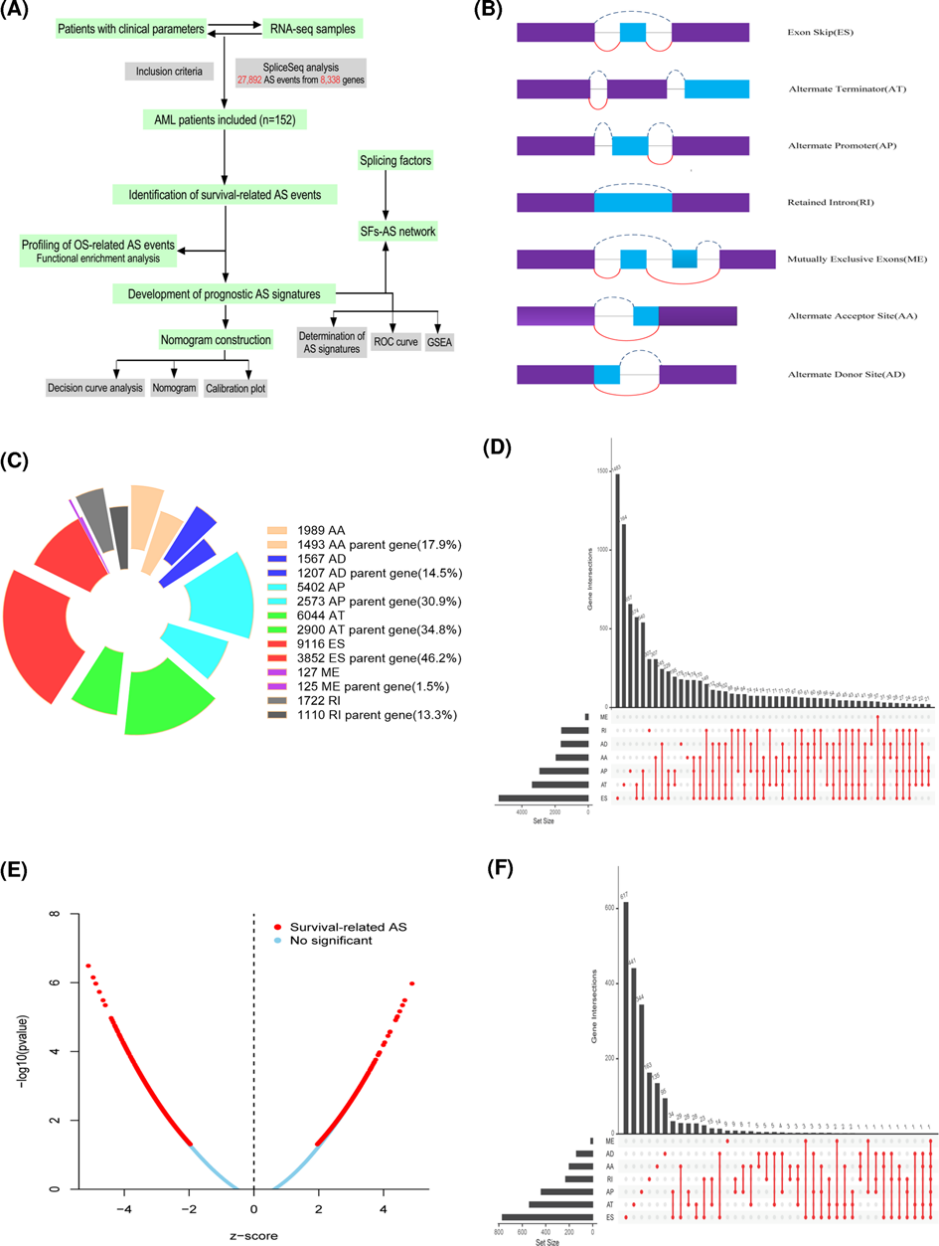
Overview of alternative splicing profiling in AML cohort (**A**) A general analysis workflow of the study. (**B**) Illustrations for seven types of AS events, including AA, AD, AP, AT, ES, ME, and RI. (**C**) Seven types of AS events and corresponding parent genes. (**D**) Upset plot of gene interactions between the seven types of the total AS events (*n* = 27,892). (**E**) Volcano plot of OS-related AS events (*n* = 3,178). (**F**) Upset plot of gene interactions between the seven types of OS-related AS events.

The general analysis workflow of our study is shown in Figure 1A. RNA splicing variant profiles were generated by SpliceSeq software. We curated 152 AML patients with AS events data and clinical information for this study. The median follow-up was 12.5 months (range 1–94 months). AS events were divided into seven types including alternate acceptor site (AA), alternate donor site (AD), alternate promoter (AP), alternate terminator (AT), exon skip (ES), retained intron (RI), and mutually exclusive exons (ME) (Figure 1B). A total of 27,892 AS events were detected in 8,338 genes, comprised 1,989 AAs in 1,493 genes, 1,567 ADs in 1,207 genes, 5,402 APs in 2,573 genes, 6,044 ATs in 2,900 genes, 9,116 ESs in 3,852 genes, 1,722 RIs in 1,110 genes, and 127 MEs in 125 genes (Figure 1C). The intersection distribution pattern of seven AS types is displayed in Figure 1D. These data showed 37 genes carrying seven types of AS events, and more than 30% of genes have greater than four types of AS events, which suggested that different combinations of splicing types jointly contribute to the transcriptome diversity. In addition, the predominant AS type in AML accounts for over 32% of all AS events.

### Identification and functional enrichment analysis of survival-related AS events

**Figure 2:**
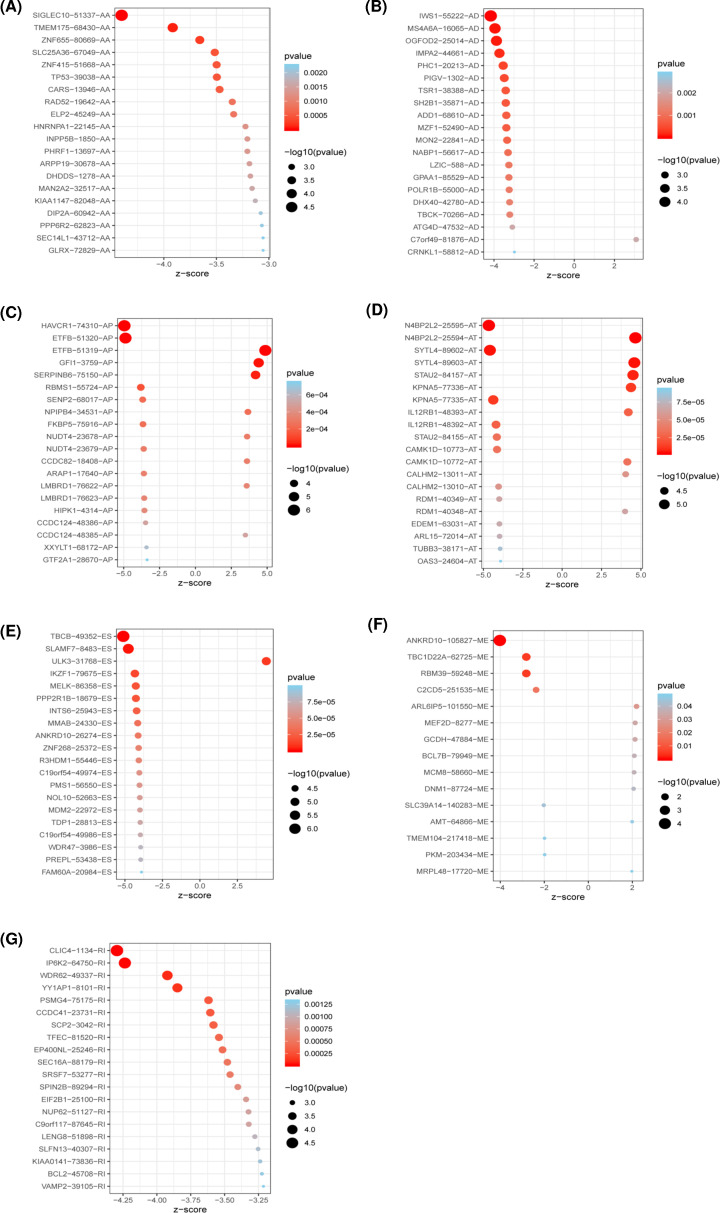
Forest plot of top 20 most significant OS-related AS events for each AS type (*P*<0.05) (**A**–**G**) *Z*-score and *P* values of top 20 overall survival associated AA, AD, AP, AT, ES, ME, and RI events, respectively. The *Z* value represents the Wald statistic, and its value is equal to the regression coefficient coef divided by its standard error se (coef), *P* values indicate whether the AS event is significantly associated with patient's outcome.

The prognostic association of each AS event with patient’s OS was determined using the univariate Cox regression analysis, and 3,178 AS events from 2,051 parent genes were found to be significantly associated with patient’s OS (*P*<0.05), accounting for 11.40% of the total AS events and 30% of total parent genes in AML (Figure 1E). Among these AS events, one gene could have more than four AS types that were related to OS (Figure 1F), such as ES, AT, AD, and AA events of the *NPEPPS* gene. Additionally, the number of each survival-related AS types were calculated, and the Wald test statistic of the top 20 most significant AS events for each AS type were displayed using forest plot (Figure 2 and Supplementary Table S1). Most of the AS events in ES, RI, AA, and AD were correlated with favorable prognostic factors.

**Figure 3:**
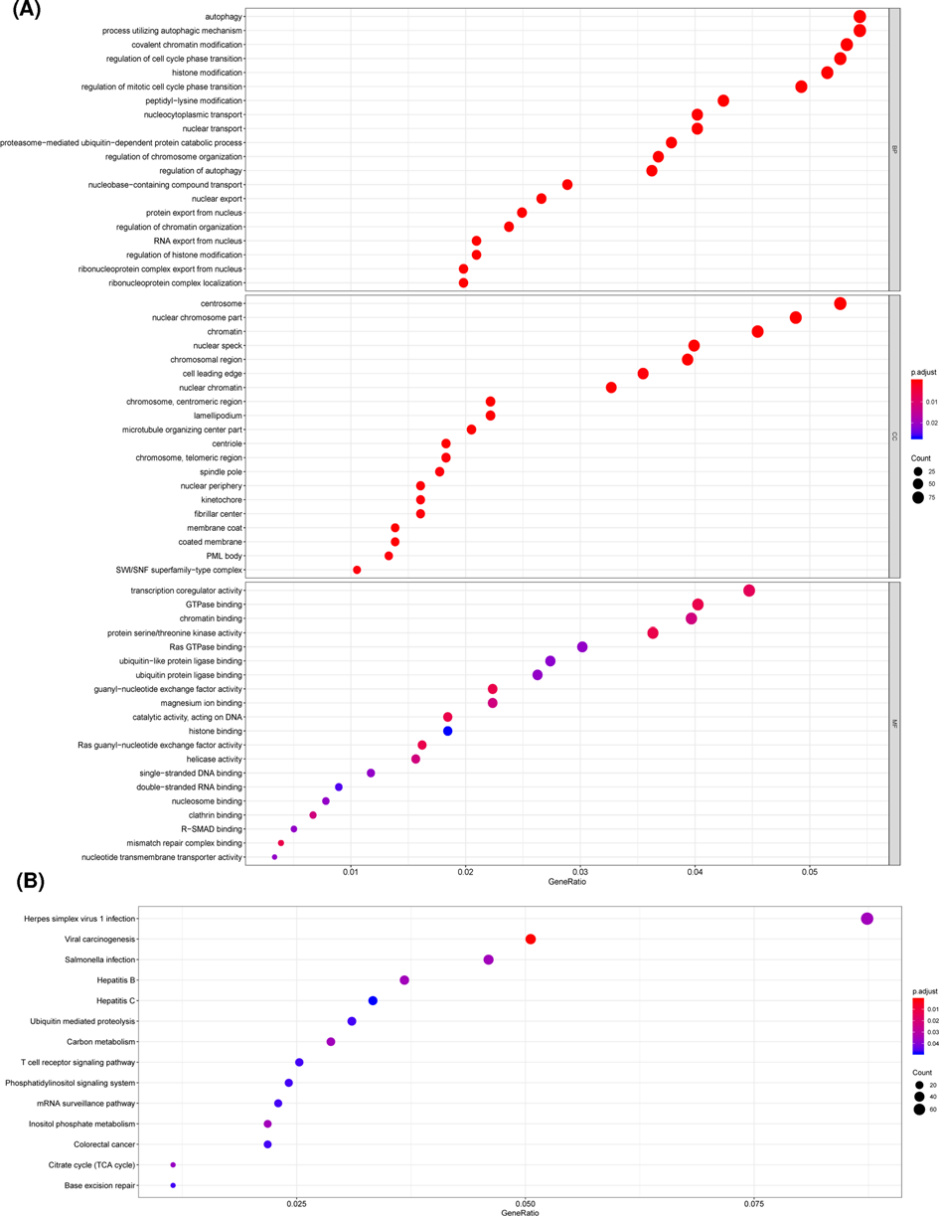
Analysis of GO terms category and KEGG pathway of the parent genes from OS-related AS events (A.djust *P* <0.05) (**A**) GO terms analysis, including biological process, cellular component, and molecular function, of OS-related AS events. (**B**) KEGG pathway of OS-related AS events.

Previous studies have revealed that alternative splicing variants could change the structures and isoform of functional proteins that are involved in the pathogenesis of various cancers [6,25]. To illuminate the potential biological functions of parent genes from these survival-related AS events in AML, all parent genes from OS-related AS events were further used for functional enrichment analysis. A total of 465 terms were found under the biological process category, highlighting the mechanisms of cell cycle control, chromatin and histone modification, autophagy, and regulation of protein assembly in aberrant splicing related patterns of AML (Figure 3A and Supplementary Table S2). Additionally, 112 terms of cellular component and 38 terms of molecular function were significant, such as ‘chromosome region’, ‘centrosome’, ‘transcription coregulator activity’, and ‘protein serine/threonine kinase activity’ (Figure 3A and Supplementary Table S2). Fourteen significant KEGG pathways were enriched, and several pathways were implicated in leukemia progression (Figure 3B), including ‘mRNA surveillance pathway’, ‘T cell receptor signaling pathway’, ‘Phosphatidylinositol signaling system’, ‘Base excision repair’, and ‘Ubiquitin mediated proteolysis’. The data suggested that these parent genes of prognostic AS events were involved in vital biological processes of AML.

### Development of the prognostic signature based on the survival-related AS events

**Figure 4:**
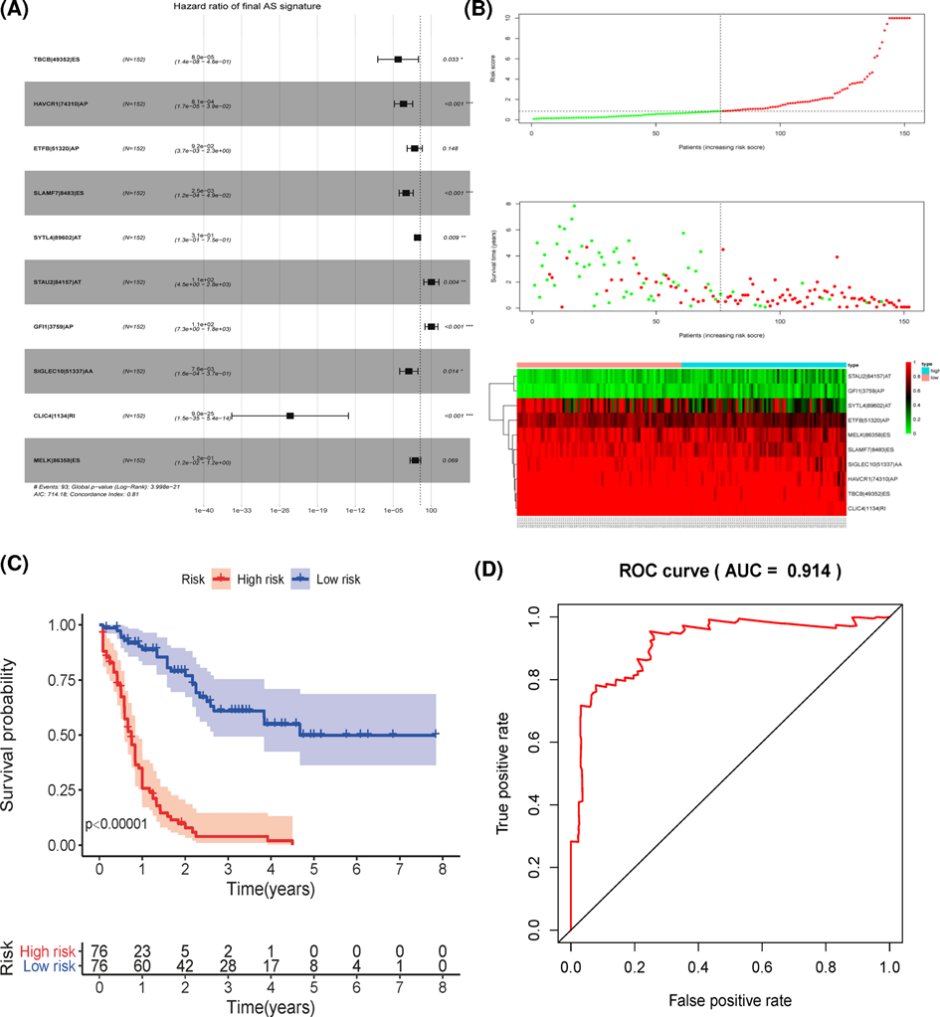
Development of the final prognostic signature based on the seven AS types (**A**) The hazard ratio of AS events in the final signature. (**B**) Distribution of patients’ risk scores, patients’ survival time, and the PSI value of the ten model AS events genes in high- and low-risk groups. (**C**) Kaplan–Meier curve of final prognostic signature built upon all seven types of AS events, in which red line indicates high-risk subgroup while blue line indicates low-risk subgroup (*P*<0.05). (**D**) The area under curve value of 3-year OS for the final signature.

Lasso penalized Cox regression model with 10-fold cross-validation was used to select the key AS events from the top significant survival-related AS events in each AS type. First, the final signature comprising 10 AS events from 7 AS types was developed using the same way, which included 1 AA event, 3 AP events, 2 AT events, 3 ES events, and 1 RI event (Figure 4A). Based on the risk score calculated by the final signature, patients were divided into high- and low-risk groups according to the median value of risk score. With the risk score of patients increased in both groups, the number of deaths was increasing (Figure 4B). Kaplan–Meier survival analysis of the final signature showed great prognostic prediction for patients in high-risk group that have significantly shorter OS than their counterparts in low-risk group (*P*<0.00001, Figure 4C). In addition, receiver operating characteristic curves were applied to assess the predictive efficiency of the signature, the final AS signature that were constructed by a specific AS type exhibited a robust and high predictive performance with the AUC value above 0.91 for the 3-year OS (Figure 4D). Then, seven prognostic signatures for each AS type were also developed using the key AS events by multivariate Cox regression analysis (Supplementary Table S3). The risk score for each patient was calculated based on the signature in each splicing type. Patients in the high-risk group defined by 7 AS signatures identified with 11 AA events, 11 AD events, 6 AP events, 7 AT events, 7 ES events, 6 ME events, and 9 RI events all have significantly worse OS than those in the low-risk group (*P*<0.00001 and Supplementary Figure S1A–G). The distribution of patient’s risk score, survival status, and the pattern of splicing variants in AS signature for each AS type are displayed in Supplementary Figure S2A-G. The area under curves (AUCs) varied in different splice type prognostic signatures, while all AUCs for the 3-year survival of patients were greater than 0.75 (Supplementary Figure S3A–G). Moreover, similar AUCs of 1-year survival for all eight prognostic signatures were observed (Supplementary Figure S4A–H).

**Figure 5:**
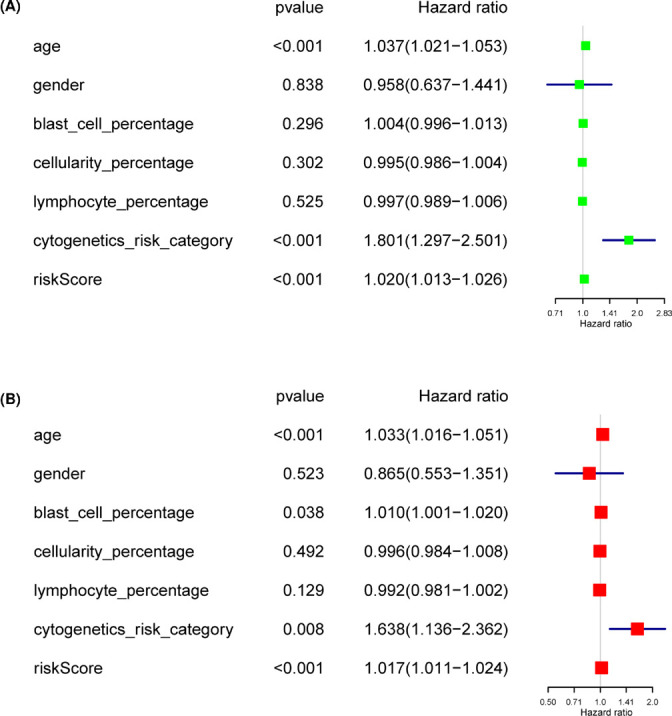
Univariate and multivariate Cox regression analysis of the risk score and clinical parameters in AML patients (*P*-value <0.05) (**A**) Univariate Cox regression analysis. (**B**) Multivariate Cox regression analysis

To further investigate the independent predictive capacity of the final signature in stratified AML patients, the univariate Cox regression analysis indicated that patients with older age, high-risk cytogenetics category, and high-risk score have a significantly shortened OS (Figure 5A). Importantly, the risk score could serve as an independent indicator after adjusting for other clinical parameters (Figure 5B), including age, gender, blast cell percentage, cellularity percentage, cytogenetics risk category, and lymphocyte percentage.

**Figure 6:**
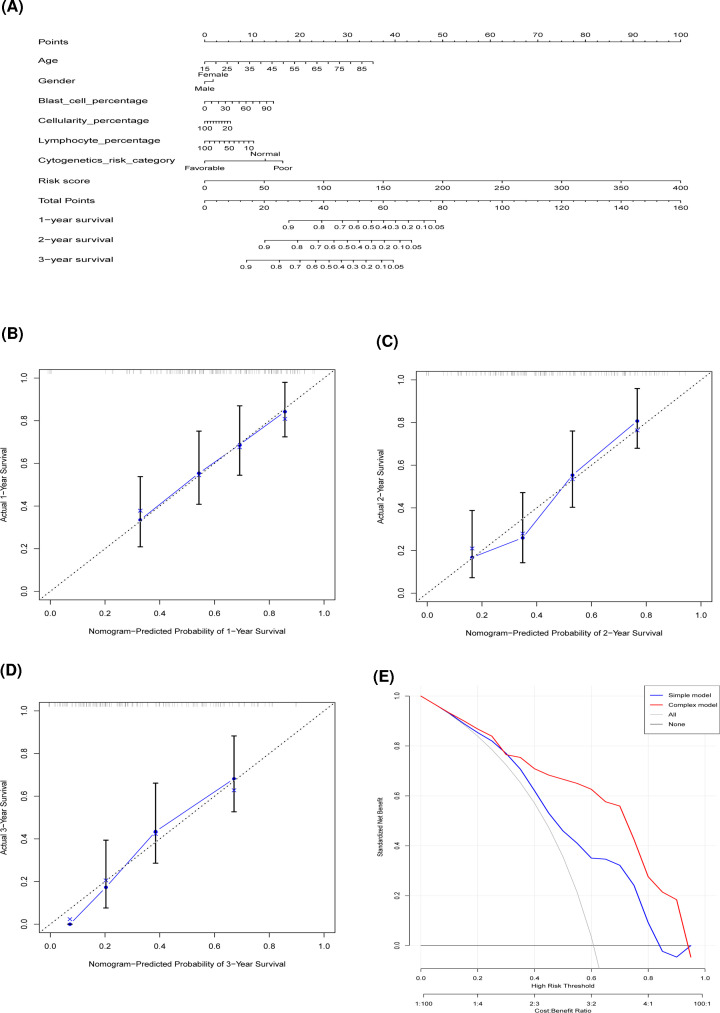
The AS-clinicopathologic nomogram for prediction on survival probability in patients with AML (**A**) Development of AS-clinicopathologic nomogram for predicting 1-, 2-, and 3-year OS for AML patients, with the final AS signature, age, gender, blast cell percentage, cellularity percentage, lymphocyte percentage, and cytogenetics risk category incorporated. (**B**–**D**) Calibration plot of the AS-clinicopathologic nomogram-predicted and observed 1-, 2-, 3-year survival in AML patients. The dashed line represents the ideal performance, and the actual performance of the final AS signature is shown by blue lines. (**E**) Decision curve analysis of the AS-clinicopathologic nomogram. The red line represents the net benefits of the final signature nomogram for predicting the OS for AML patients. The blue line stands for treat-all scheme varying with threshold probability, while the black line represents the net benefit of treat-no scheme.

To provide a quantitative method to predict the individualized survival probability of AML patients, a nomogram integrating the risk score of the final signature and clinical factors was constructed (Figure 6A). The actual and predicted performance of the nomogram for 1, 2, and 3 years in AML patients showed high predictive accuracy (Figure 6B–D). Decision curve analysis (DCA) indicated that patients with AML can benefit from the prediction by the final signature (Figure 6E).

### GSVA and GSEA of the final signature based on 10 AS events

**Figure 7:**
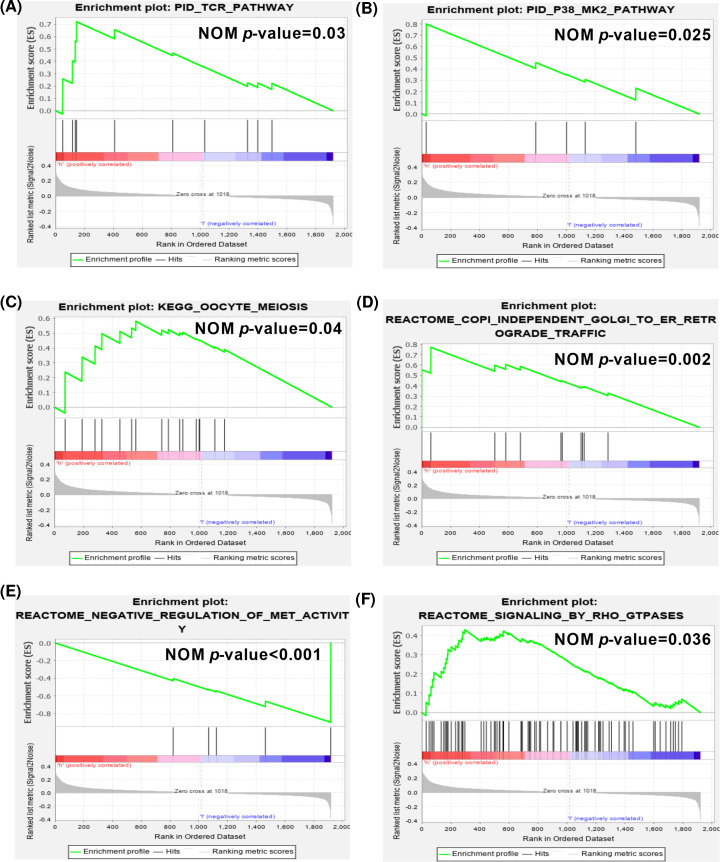
Gene set enrichment analysis of the parent genes of OS-related AS events in high- and low-risk groups (**A**) PID_TCR pathway. (**B**) PID_P38_MK2 pathway. (**C**) KEGG_oocyte meiosis. (**D**) Reactome_COPI_Independent golgi to ER_retrograde traffic. (**E**) Reactome_negative regulation of met_activity. (**F**) Reactome_signaling by RHO GTPases.

To further throw light on the differential functional gene sets in AML, gene set variation analysis was performed, and 15 activated GO terms were significantly enriched in MSigDB_C5_GO (Table 1) and 10 significantly pathways were found in C2 curated gene sets (Table 2), such as ‘co-stimulation by the CD28 family’, ‘RADMACHER AML prognosis’, and ‘REACTOME FLT3 signaling’. Furthermore, patients with high risk score predicted by the signature have a worse prognosis. Gene set enrichment analysis was applied to investigate the potential pathways (Figure 7). ‘TCR pathway’, ‘P38_MK2 pathway’, ‘KEGG oocyte meiosis’, and ‘Rectome signaling by RHO GTPases’ were found enriched in the high-risk group, these were consistent with the results of functional enrichment and the findings of GSVA mentioned above. The CD28 family of receptors, which are key members of the immunological synapse, including CD28, CTLA-4, ICOS, and PD-1, are able to deliver co-stimulatory or inhibitory signals on T cells through interacting with their ligands [26]. It may indicate that tumor cells can exploit regulators, such as CTLA-4, and PD-1, that involved in above identified pathways to engage T cells to generate immunosuppressive microenvironment, which contributed to the pathogenesis and progression of AML.

**Table 1:** Differentially expressed GO terms based on GSVA analysis of parent genes from survival-related AS events from C5 GO terms in MSigDB database between high- and low-risk groups patients (Adj. *P* <0.05)

GO terms	LogFC	AveExpr	*t*	*P*.Value	Adj.*P*.Val	*B*
GO_REGULATION_OF _CELLULAR_KETONE _METABOLIC_PROCESS	-0.1644	-0.0018	-4.4947	1.37E-05	0.0092	2.9358
GO_NEGATIVE_REGULATION _OF_TRANSMEMBRANE_RECEPTOR_ PROTEIN_SERINE_THREONINE_ KINASE_SIGNALING _PATHWAY	0.1655	-0.0095	4.0244	8.99E-05	0.0201	1.2556
GO_INTERFERON _GAMMA_PRODUCTION	-0.2014	0.0003	-3.9483	0.0001	0.0201	0.9973
GO_NEGATIVE_REGULATION _OF_CELL_MOTILITY	-0.1234	-0.0107	-3.9375	0.0001	0.0201	0.9611
GO_NEGATIVE_ REGULATION_OF _LOCOMOTION	-0.1256	-0.0122	-3.9227	0.0001	0.0201	0.9112
GO_PROTEIN_ TYROSINE_KINASE _BINDING	-0.1509	0.0073	-3.8792	0.0002	0.0210	0.7662
GO_NEGATIVE_ REGULATION_OF_ CELLULAR_RESPONSE_ TO_GROWTH_FACTOR_ STIMULUS	0.1302	-0.0102	3.7849	0.0002	0.0249	0.4563
GO_SMAD_ PROTEIN_SIGNAL _TRANSDUCTION	0.1765	-0.0002	3.7834	0.0002	0.0249	0.4513
GO_HOMOTYPIC_ CELL_CELL_ ADHESION	-0.2023	0.0095	-3.7388	0.0003	0.0251	0.3071
GO_POSITIVE_ REGULATION_OF _JUN_KINASE_ ACTIVITY	-0.1894	0.0228	-3.5808	0.0005	0.0339	-0.1929
GO_POSITIVE _REGULATION_OF _PEPTIDE_SECRETION	-0.1238	-0.0138	-3.5071	0.0006	0.0377	-0.4200
GO_ENZYME _REGULATOR_ ACTIVITY	-0.0738	-0.0036	-3.4813	0.0007	0.0377	-0.4988
GO_DNA_BINDING _TRANSCRIPTION_ FACTOR_ACTIVITY	0.0855	0.0042	3.4803	0.0007	0.0377	-0.5017
GO_TRANSFORMING _GROWTH_FACTOR_ BETA_RECEPTOR_ SIGNALING_PATHWAY	0.1060	-0.0036	3.4716	0.0007	0.0378	-0.5280
GO_POSITIVE _REGULATION_OF_ T_CELL_PROLIFERATION	-0.1685	0.0062	-3.3835	0.0009	0.0427	-0.7920

**Table 2:** Differentially expressed pathways based on GSVA analysis of parent genes from survival-related AS events from C2 curated gene sets in MSigDB database between high- and low-risk groups patients (Adj. *P* <0.05)

Pathway	LogFC	AveExpr	*t*	*P*.Value	Adj.*P*.Val	*B*
REACTOME_COSTIMULATION _BY_THE_CD28_ FAMILY	-0.2840	-0.0205	-4.8911	2.51E-06	0.0036	4.4716
PARK_HSC_AND_ MULTIPOTENT_ PROGENITORS	-0.1900	-0.0063	-4.3184	2.81E-05	0.0136	2.2952
PECE_MAMMARY_ STEM_CELL_DN	0.2058	0.0063	4.1734	5.01E-05	0.0162	1.7769
REACTOME_METABOLISM _OF_VITAMINS_AND_ COFACTORS	-0.1409	-0.0020	-4.1459	5.58E-05	0.0162	1.6802
KUMAR_TARGETS _OF_MLL_AF9_ FUSION	-0.1118	0.0076	-4.0506	8.09E-05	0.0168	1.3488
ELVIDGE_ HYPOXIA_DN	-0.1538	-0.0008	-3.9581	0.0001	0.0208	1.0331
RADMACHER_AML _PROGNOSIS	-0.1746	-0.0032	-3.9290	0.0001	0.0208	0.9350
REACTOME_FLT3 _SIGNALING	-0.1127	-0.0058	-3.5445	0.0005	0.0302	-0.3057
SANSOM_APC _TARGETS_DN	-0.1736	-0.0105	-3.4294	0.0008	0.0365	-0.6564
REACTOME_MAPK6 _MAPK4_SIGNALING	-0.1840	-0.0218	-3.2583	0.0014	0.0494	-1.1593

### Identification of regulatory relations between splicing factors (SFs) and AS event

**Figure 8:**
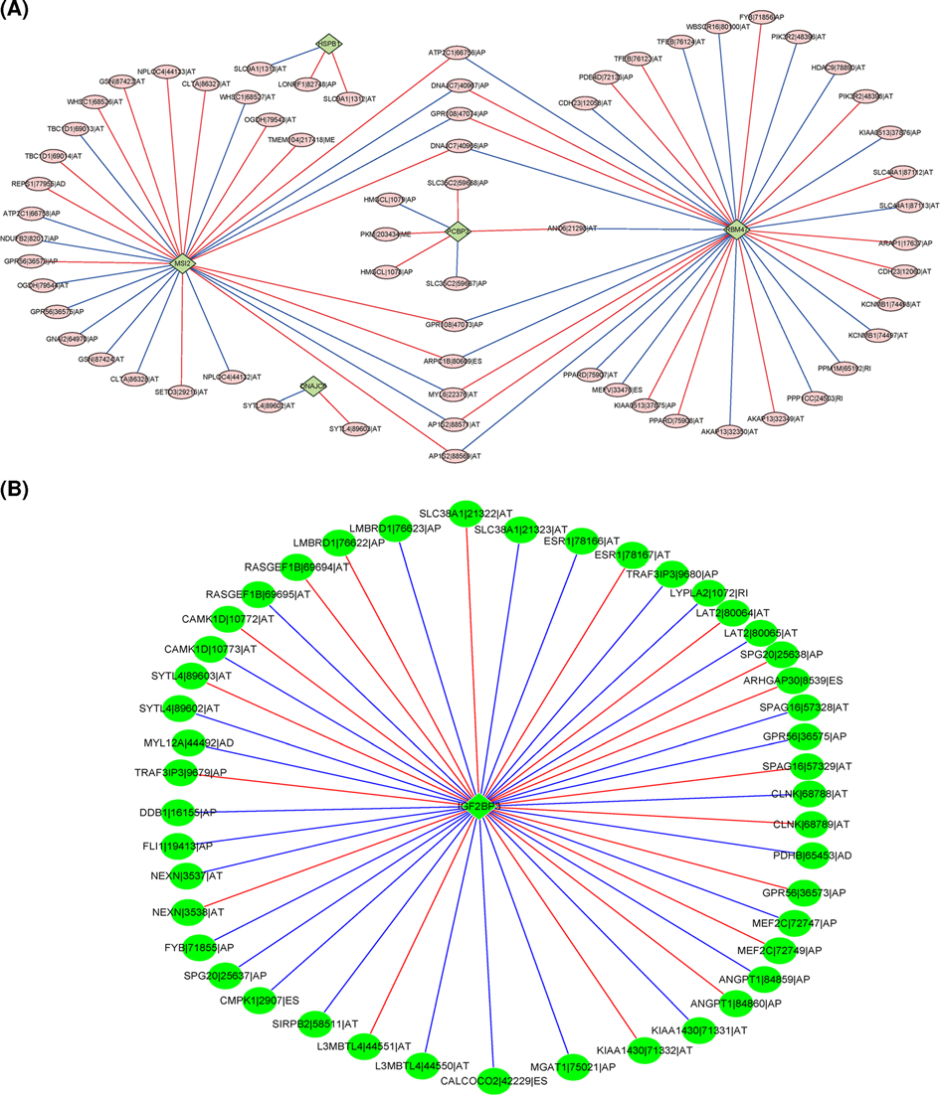
The correlation network of the SFs with OS-related AS events (**A**) The correlation network of the expression of SFs with the PSI values of OS-related AS events. Rhombus represents the SFs, and circle represents AS events. In addition, red line represents the positive correlation between SFs and AS events, blue line stands for the negative correlation between SFs and AS events. (**B**) The correlation network of the expression of OS-related SFs with the PSI values of OS-related AS events. Rhombus represents the survival-related SFs, and circle represents AS events. In addition, red line represents the positive correlation between SFs and AS events, blue line stands for the negative correlation between SFs and AS events.

It is well-recognized that dysregulated AS events were mediated by several SFs. The correlation analysis was performed to assess the correlation between SFs expression and the PSI scores of survival-related AS events with the coefficient greater than 0.4 as cut-off value (*P*<0.001), 15 SFs were identified to be significantly associated with OS-related AS events. HSPB1, MSI2, RBM47, PCBP3, and PCBP4 ranked as the top 5 SFs according to the node number equal or more than 15 (Figure 8). MSI2, RBM47, and PCBP3 were significantly higher expressed in patients than that in normal cases, while HSBP1 had decreased expression (Supplementary Figure S5). To determine which SF was associated with the patient’s survival, 68 SFs were found to be significantly associated with AML patients’ OS using univariate Cox regression analysis based on gene expression (*P*<0.05, Supplementary Table S4). Of these OS-related SFs, high expression of RBM47 (HR = 1.0986, *P*=0.0293) was associated with an unfavorable prognosis, while increased expression of PCBP3 (HR = 0.9238, *P*=0.0219) was linked with prolonged survival. Furthermore, correlations between the PSI values of OS-related AS events and the expression of OS-related SFs were investigated, only IGF2BP3 was found to be associated with 45 AS events. In addition, increased expression of IGF2BP3 markedly predicted shortened OS (HR = 1.3650, *P*=3.64E-5). As an RNA-binding protein, previous studies demonstrated that IGF2BP3-mediated targeting of oncogenic transcripts of Myc and CDK6 promotes hematopoietic progenitor proliferation in MLL-rearranged B-ALL [27]. In addition, CDK6 is a direct target of MLL fusion proteins and plays an important role in the proliferation of MLL-rearranged leukemia [28]. MLL-fusions could lead to an aggressive acute myeloid leukemia. These data suggested that IGF2BP3 with its RNA-binding partners may serve as a potential therapeutic target in AML disease through interacting with CDK6.

## Discussion

Alternative splicing events represent a vital molecular regulatory mechanism in modifying mRNA isoforms that can generate a diversity of mRNA and proteins with different regulatory and functional properties [7]. Previous studies have indicated that the plasticity of AS events can be deliberately exploited by cancer cells to produce the aberrant changes at different levels, such as the altered activity and expression abundance of the genes that contribute to cancer cell survival, proliferation, migration, and therapeutic resistance [29–31]. Studies have suggested that aberrant AS events are implicated in cancer development [30]. The large-scale RNA sequencing data in publicly database has made it easy to investigate the AS events that occurr in various cancers. Several studies in the investigation of AS events have revealed that alternative spliced variants and cancer-specific splicing variants could be identified as potential diagnostic and prognostic biomarkers in different cancers [32–34]. For example, Zhen Zong et al. proposed a prognostic signature for risk stratification in colorectal cancer based on alternative splicing profiling [35]. AML is a fetal hematologic malignancy characterized by uncontrollable clonal disorder of the myeloid cells. Although treatment advances have extended the survival of younger patients, the prognosis of older patients with AML, who account for the majority of new cases, remains poor. Thus, there is an urgent need to identify novel prognostic and therapeutic biomarkers to monitor disease development. The abnormalities in AS events in AML progression and drug resistance have attracted interests as several studies identified mutations in splice factors can serve as important drivers of hematological malignancies [36,37]. However, the prognostic significance of AS events in AML is unclear.

In the present study, we performed a systematic identification and analysis of survival related AS events in 152 patients with AML from TCGA portal. About 3,178 AS events were significantly associated with OS. Clinically, survival benefit was found in all the seven alternative splicing patterns. Specifically, one gene can generate several mRNAs that result in different transcripts and various protein isoforms with opposing functional effects. Interestingly, most top 20 survival-related AS events of seven splicing types tend to have a favorable prognosis. Additionally, some parent genes harboring different AS events have opposite prognostic effects for patients (Supplementary Table S1), for example, the ES variant type of ADD1 gene had a protective effect on the patient’s survival, while the AP type predicted unfavorable survival. Even the AS type at different locations in the same gene showed the opposite effect on survival. Functional enrichment analysis revealed that these parent genes were involved in several pathways that have been demonstrated to mediate leukemia progression such as T-cell receptor signaling [38] and prevent the production of potentially toxic proteins from aberrant mRNA translation events [39]. It is not surprisingly that some parental genes of survival-related AS-events are enriched in a number of viral/infection related GO terms. This might be due to AS event is one of the main adaptive protection mechanisms against external intrusions through maintaining protein diversity. Acute myeloid leukemia is a type of aggressive blood malignancies that diverse immune-related responses are involved in the disease progression, including immune response to infection. In addition, a large number of studies have found that changes in AS events are related to cancer and many other diseases. It is estimated that 300 splicing-related genes are mutated in all types of cancer from the International Cancer Genome Consortium (ICGC), several hnRNP family members, SR proteins and along with SR-protein kinases, and RBM proteins are the most frequently mutated genes [40]. The prognostic genes we identified included FLT-3, IDH1, TP53, BCL2, SRSF1, CD44, RBM4, STAT3, hnRNPU, and hnRNPL, which was consistent with previous studies that these genes play critical roles in leukemogenesis and drug resistance through altered splicing of tumor suppressor, oncogenes and dysregulation of the apoptotic signaling pathways [36].

AS events in cancers were considered as a great untapped potential in monitoring patient’s survival when compared with the transcriptome-level analysis. The PSI value, a ratio between reads including or excluding exons, makes it possible for calculating seven types of AS events within tumors. Combined with follow-up data, the predictive model was constructed based on each type of AS pattern. The model comprised of alternate donor site (AD) events showed higher distinguishing capacity for predicting survival of AML patients than the classifiers built with other six types of AS events. Furthermore, the combination of all seven types of AS patterns could promote to identify a better prognostic predictor, the final OS-related AS signature showed a robust and markedly improved performance with the AUC above 0.91. This suggested that AS events could be applied to predict the prognosis for AML patients. Xie ZC et al. recently proposed prognostic alternative splicing regulatory network based on the AS events profiling of AML [41]; however, it mainly focused on the splicing network of AS events and splicing factors, and the predictive efficacy of alternate terminator model (AUC = 0.781) is inferior to our final AS signature in terms of ROC analysis. The predictive independency of the final signature can be influenced by some clinical parameters. For example, older age is an unfavorable factor for AML patients, while the signature still had significant stratification irrespective of other clinical parameters. Additionally, we supposed that the model combining our final signature and important clinical factors may present a more reliable prediction efficacy for speculating patient’s survival. The prognostic nomogram integrated with age, cytogenetics risk category, and the final signature provided individualized survival risk assessment. We applied DCA analysis, a novel statistical approach of calculating the net benefit against a range of threshold probabilities [42], to assess whether nomogram would help to improve patient outcome. Obviously, the decision curves indicated that our final AS-clinicopathologic nomogram to predict survival probabilities adds more benefit than all or none of the patients were treated.

Furthermore, differential biological processes and pathways enriched in low- and high-risk group stratified by the final 10 AS-event signature were investigated by GSVA and GSEA algorithms. Similar cancer-related pathways that found by functional enrichment analysis were observed. Several leukemia-related specific pathways [43–45], including co-stimulation by the CD28 family, HSC and multipotent progenitors, targets of MLL-AF9 fusion pathway, AML prognosis, TCR pathway, P38 and MK2 pathway, and FLT3 signaling, which were demonstrated to play crucial roles in the regulation of leukemogenesis and progression. Moreover, biological pathways that contribute to tumorigenesis, such as invasiveness, migration, and proliferation, were identified. The exact underlying mechanisms of the AS signature need further validation and may provide valuable therapeutic targets for AML treatment.

As the key regulatory elements of AS events, splicing factors (SFs) recognized and bind to cis-regulatory elements during pre-mRNA process. Aberrant alternations in SFs expression have been observed in tumors [30,46] SFs contribute to tumorigenesis via regulating AS events and serving as oncogenes or pseud-oncogenes [47]. In the present study, the potential correlation network between SFs and survival-associated AS events revealed that RBM47, MSI2, PCBP3, HSBP1, and DNAJC6 were key SFs that may be involved in the regulation of AS events process in AML progression. Indeed, it is consistent with previous evidence. RBM47 has been demonstrated to promote transforming growth factor-β (TGF-β)-induced EMT by alternative splicing of the exon 20 of TJP1 in lung cancer cells [12], and suppress breast cancer progression through altering splicing of a subset of its target mRNAs, such as dickkopf WNT signaling pathway inhibitor 1 [48]. In addition, RBM47 elevated IL-10 expression and enhanced the immunosuppression of B cells [49]. MSI2 has been shown to be mainly expressed in hematopoietic stem cells, and it markedly regulates normal hematopoiesis and promote aggressive myeloid leukemia [50]. Additionally, aberrant expression of PCBP3 and HSBP1 were significantly associated with cancer development [51,52] and therapy resistance [53]. The results suggested that these SFs may have critical roles in AML. Furthermore, among 68 survival-related SFs, high expression of PCBP3 and decrease RBM47 expression predicted favorable survival for AML patients. The correlation network of survival-related SFs and AS events identified IGF2BP3 as the only SF that correlated with AS events, and most important, IGF2BP3 has been clinically relevant in leukemia, while its specific molecular mechanism in AML has not been clearly deciphered [27].

Our current study provided a systematic analysis of AS events and developed risk prognostic risk signatures based on the survival-related AS events in AML patients, while several limitations should be taken into consideration when interpreting the findings. There are a relatively small number of AML patients enrolled in this study, and no normal cases available for comparison analysis. The prognostic utility of survival-related signature needs the independent external validation, while no accessible data set available. Importantly, the present study was conducted based on publicly accessible high-throughput RNA sequencing data, and therefore, experimental and clinical verification are warranted in further investigations.

In summary, we performed a comprehensive analysis on profiling the AS events in AML patients, developed a robust survival-related AS signature for predicting patient’s outcome, and highlighted the key splicing factors that tightly correlated with survival-related AS events. This might contribute to monitor the patient' prognosis and provide novel clues for targeted molecular implications.

## Supplementary Material

Supplementary Figures S1-S5

Supplementary Tables S1-S4

## Data Availability

The data analyzed in this study are available in the following repositories: 1. TCGA: https://portal.gdc.cancer.gov/. 2. TCGA SpliceSeq: https://bioinformatics.mdanderson.org/TCGASpliceSeq.

## Abbreviations

AA: acceptor site
AD: alternate donor site
AIC: akaike information criterion
AP: alternate promoter
AS: alternative splicing
AT: alternate terminator
AUC: area under curve
BP: biological process
CC: cellular component
DCA: decision curve analysis
EMT: epithelial–mesenchymal transition
ES: exon skip
GSEA: gene set enrichment analysis
GSVA: gene set variation analysis
KEGG: Kyoto Encyclopedia of Genes and Genomes
ME: mutually exclusive exons
MF: molecular function
MSigDB: Molecular Signatures Databases
OS: overall survival
PSI: percent spliced in
RI: retained intron
ROC: the receiver operating characteristics curve
SFs: splicing factors
TCGA: The Cancer Genome Atlas
