# Supplementary material for: Identification of survival-related alternative splicing signatures in acute myeloid leukemia
Source: Biosci Rep. 2021 Jul 20;41(7):BSR20204037. doi: 10.1042/BSR20204037 (PMC8292762; doi:10.1042/BSR20204037)
Supplement: Supplementary Figures S1-S5 [file BSR-2020-4037_supp.pdf]

Supplementary materials

Supplementary Figures and Legends

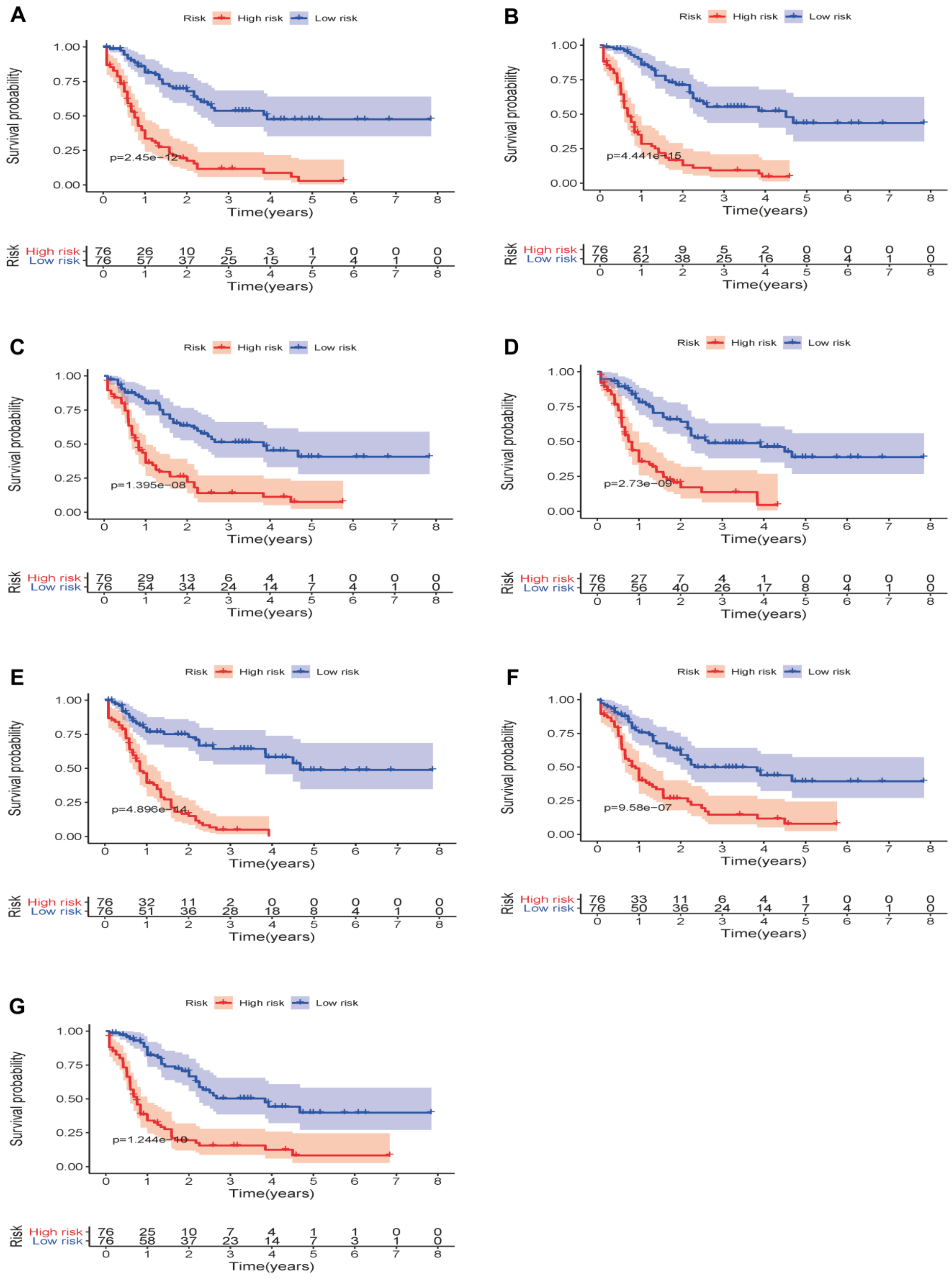

Supp Figure 1. Kaplan-Meier plots of prognostic signatures built with each type of AS event for AML patients ($P < 0.05$). (A-G). Kaplan-Meier plot of prognostic signature built with AA, AD, AP, AT, ES, ME, and RI events, respectively. Red line indicates high-risk subgroup while blue line indicates low-risk subgroup.

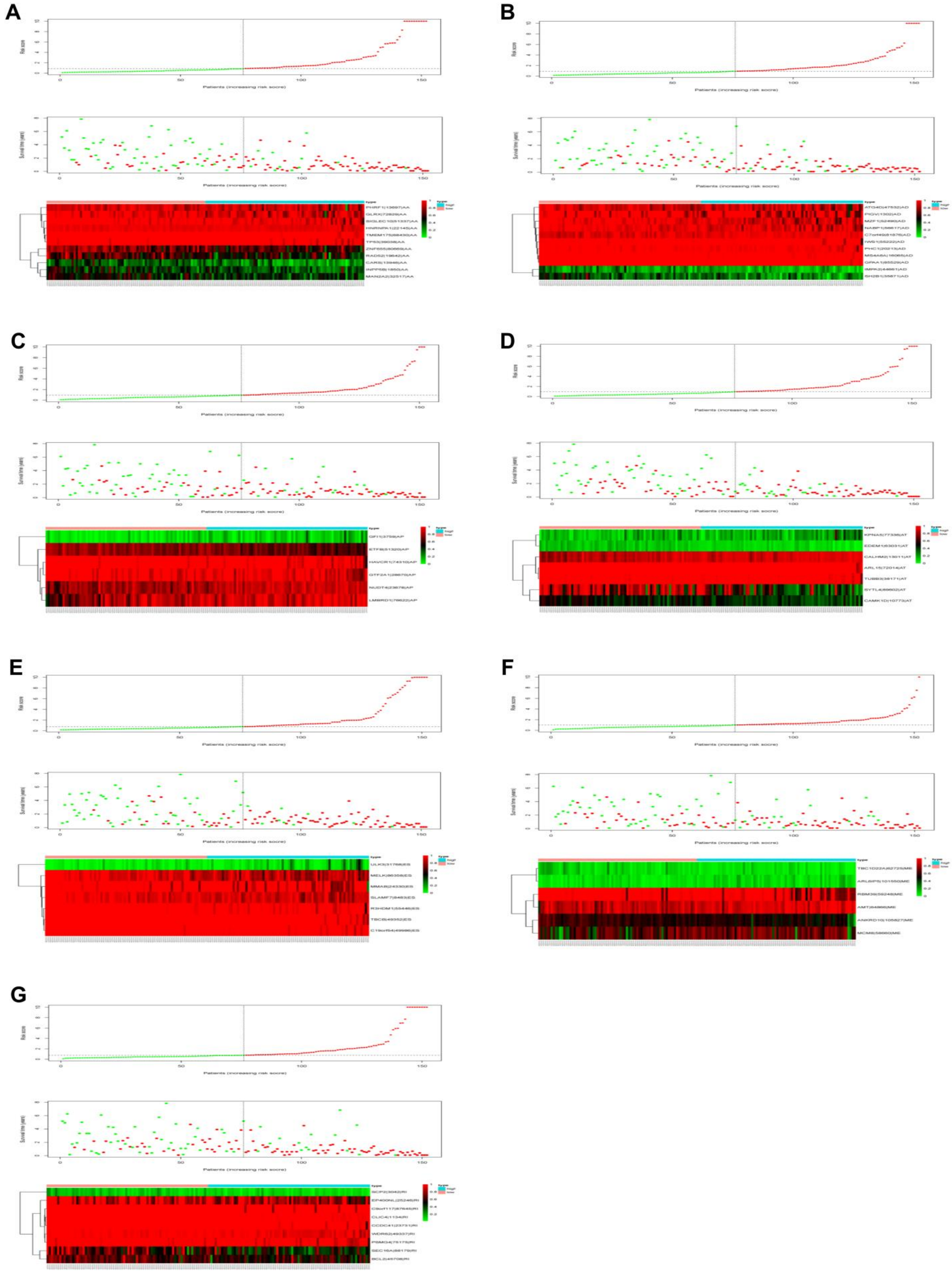
A
B
C
D
E
F
G

Supp Figure 2. Development of the prognostic signature based on the PSI values of the OS-related AA, AD, AP, AT, ES, ME, and RI events. **(A-G)**. Determination and analysis of the prognostic AS signatures in AML cohort. AML patients were divided into high- and low-risk subgroups based on the median cut of risk score calculated separately. The upper part of each assembly indicates distribution of patients' survival status and survival times ranked by risk score, the middle part represents the risk score curve, and the bottom heatmap displays splicing pattern of the AS signature from each AS type. Color transition from blue to red indicates the increasing PSI score of corresponding AS event from low to high.

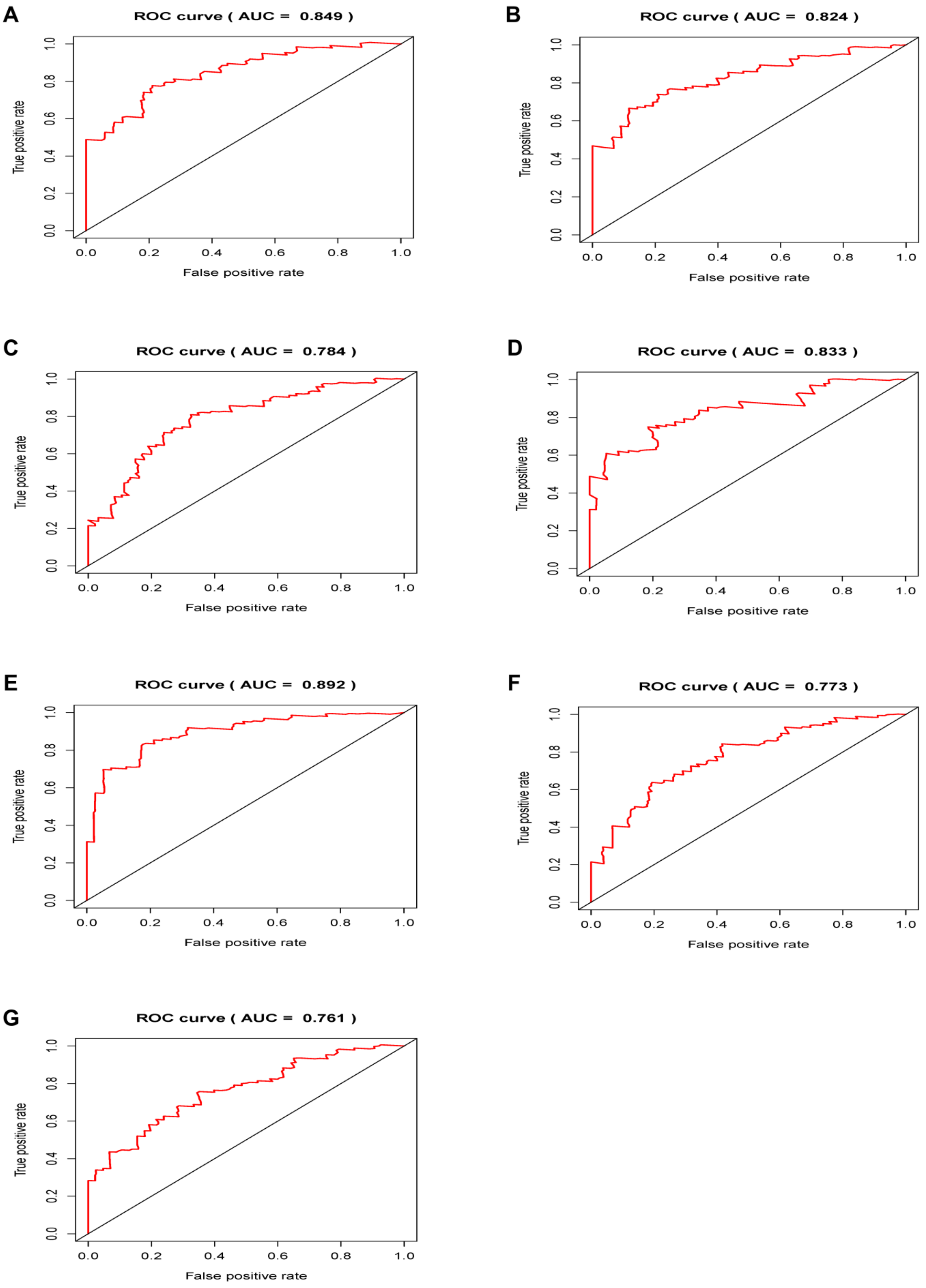
A
ROC curve (AUC = 0.849)
True positive rate
False positive rate
B
ROC curve (AUC = 0.824)
True positive rate
False positive rate
C
ROC curve (AUC = 0.784)
True positive rate
False positive rate
D
ROC curve (AUC = 0.833)
True positive rate
False positive rate
E
ROC curve (AUC = 0.892)
True positive rate
False positive rate
F
ROC curve (AUC = 0.773)
True positive rate
False positive rate
G
ROC curve (AUC = 0.761)
True positive rate
False positive rate

Supp Figure 3. ROC curves with calculated area under curves (AUCs) of prognostic signatures constructed by each type of AS event in AML cohort for risk prediction in 3 years.

(A-G). The curves of time-dependent AUCs versus time (3-year) of prognostic signature constructed by AA, AD, AP, AT, ES, ME, and RI events, respectively.

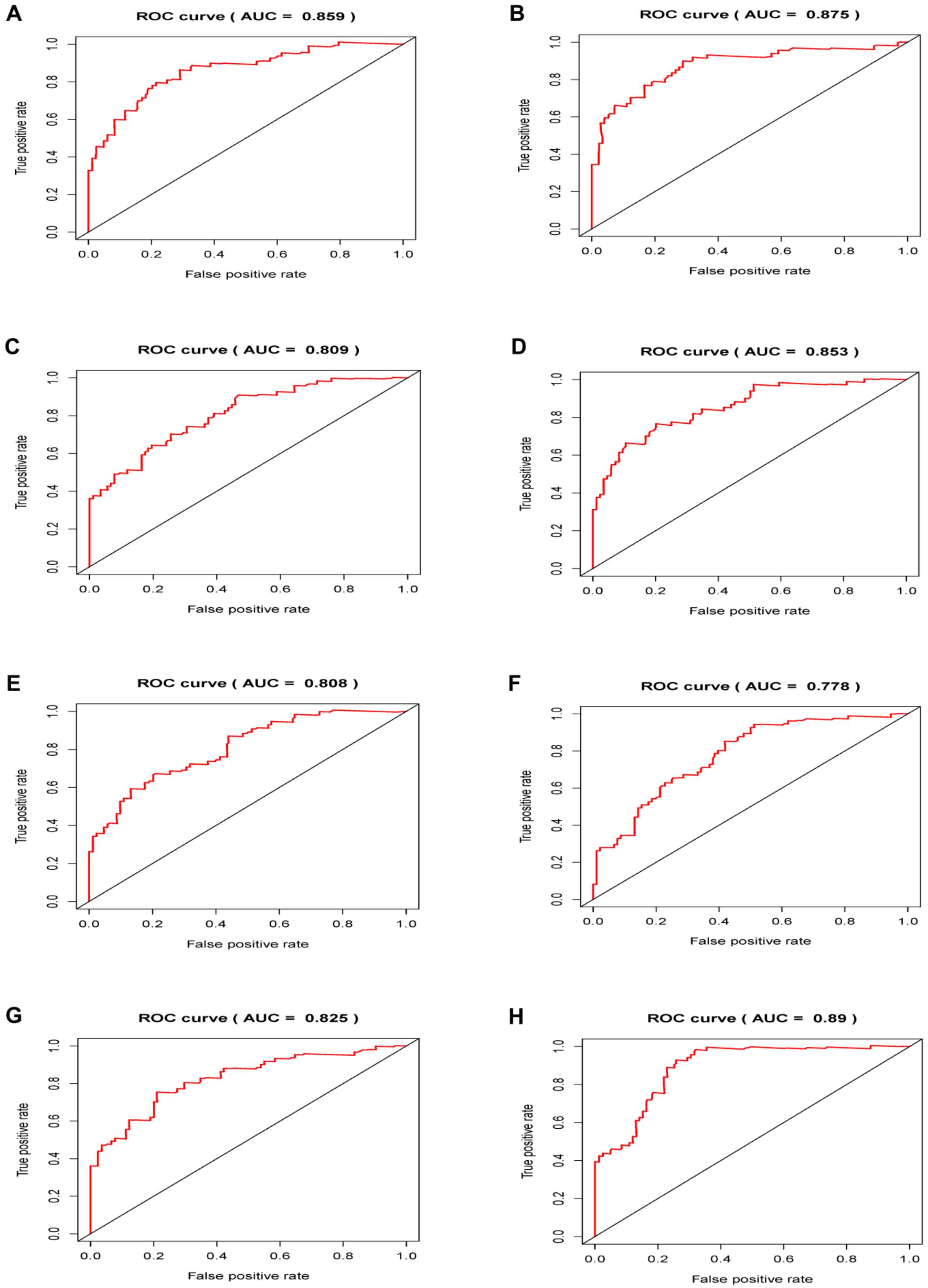
A
ROC curve (AUC = 0.859)
B
ROC curve (AUC = 0.875)
C
ROC curve (AUC = 0.809)
D
ROC curve (AUC = 0.853)
E
ROC curve (AUC = 0.808)
F
ROC curve (AUC = 0.778)
G
ROC curve (AUC = 0.825)
H
ROC curve (AUC = 0.89)
True positive rate
False positive rate
0.0
0.2
0.4
0.6
0.8
1.0

Supp Figure 4. ROC curves with calculated area under curves (AUCs) of prognostic signatures constructed by either one type or all seven AS types in AML cohort for risk prediction in 1 years. (A-G). The curves of time-dependent AUCs versus time (1-year) of prognostic signature constructed by AA, AD, AP, AT, ES, ME, and RI events, respectively. (H). The curves of time-dependent AUCs versus time (1-year) of the final AS signature.

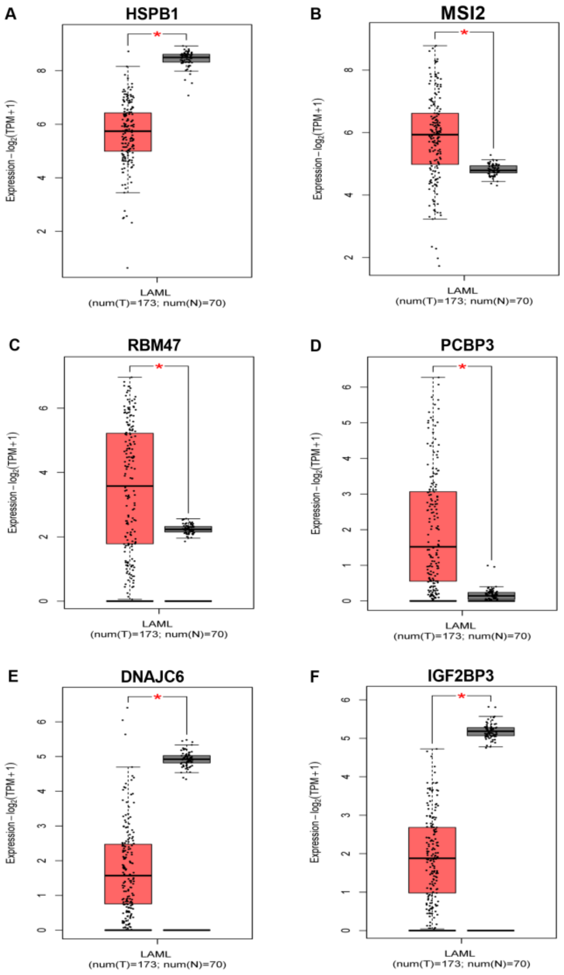
A
HSPB1
B
MSI2
C
RBM47
D
PCBP3
E
DNAJC6
F
IGF2BP3
Expression - log2(TPM + 1)
LAML
(num(T)=173; num(N)=70)

Supp Figure 5. The gene expression of top five correlated SFs in AML patients and normal cases (GEPIA database, $P < 0.05$) **(A).** HSBP1; **(B).** MSI2; **(C).** RBM47; **(D).** PCBP3; **(E).** DNAJC6; **(F).** IGF2BP3
